# Effect of professional commitment on the career plans of intern doctors

**DOI:** 10.12669/pjms.41.2.10253

**Published:** 2025-02

**Authors:** Sema Turan, Serhat Hayme

**Affiliations:** 1 Sema Turan, MD Erzincan Binali Yıldırım University Faculty of Medicine, Department of Public Health, Erzincan/ Turkiye; 2 Serhat Hayme, PhD Erzincan Binali Yıldırım University Faculty of Medicine, Biostatistics and Medical Informatics Department, Erzincan, Turkiye

**Keywords:** Career plan, Intern doctor, Medical student, Professional commitment

## Abstract

**Objective:**

The career choices of medical school graduates are extremely important because of their impact on a country’s medical workforce planning. This study aimed to investigate the impact of medical students’ level of professional commitment (PC) on their career plans.

**Method:**

This cross-sectional study was conducted between April 15^th^ and November 15^th^ 2023, in a medical faculty in Erzincan Province in eastern Turkiye. A 16-question questionnaire and the medical students’ PC scale were used. A total of 129 intern doctors (93.5%) participated in the study. The t test, Mann-Whitney U test, and Kruskal-Wallis test were used in the statistical analyses.

**Results:**

Students who voluntarily chose medical school, aimed to become specialist doctors, selected specialties with higher occupational risks, and intended to work in Turkiye after obligatory service exhibited higher levels of PC. Notably, those inclined towards family practice specialty demonstrated lower levels of PC, whereas public health specialty, basic sciences, and certain clinical sciences were not preferred by any of the students.

**Conclusion:**

The results obtained suggest that PC levels may influence the career plans of medical students. Further research into factors affecting changes in PC throughout medical education and their impact on future career goals will enhance understanding in this area.

## INTRODUCTION

The career choices of medical school graduates are extremely important because of their impact on a country’s medical workforce planning, and thus on the protection and improvement of public health.^1^ The numerical shortage and unbalanced dispersion of doctors in Turkiye is an ongoing problem. According to 2022 data, the number of doctors per 100,000 people in Turkiye is 228, ranking last among The Organization for Economic Cooperation and Development countries. Of the total number of 194,688 doctors, 53,697 are general practitioners, 95,600 are specialists, 42,687 are undergoing medical residency and 2,704 are undergoing subspecialty training.^2^

Medical education in Turkiye lasts six years (seven years if there is a preparatory period in English).^3^ Specialists and general practitioners who complete their education in the field in which they are qualified according to the results of the exam complete their obligatory service (300–600 days) in units affiliated with the Turkish Ministry of Health. After completing their obligatory service, they work in the public or private sector according to what they wish to do.^4^ Medical education is a demanding process that requires long-term endeavour and commitment.^5^ Professional commitment (PC) is defined as a commitment to professional goals, values, beliefs, and a desire to continue in a profession.^6^ Individuals with high PC demonstrate their abilities to the best of their abilities, make an effort to advance their careers, and are less likely to quit.^5^

PC has three main components, comprising affective, continuance, and normative commitment. Affective commitment refers to an individual’s positive attitude toward their profession and their willingness to continue working in that profession based on their emotional motivation. Individuals who have a strong emotional connection with their profession have the chance to advance in their profession and improve their professional skills. Continuance commitment refers to the act of perseverance in an occupation when faced with the decision to leave or stay. Finally, normative commitment refers to an individual’s strong sense of duty and integration of professional regulations into personal identity.^7^

Studies have shown that many factors are influential in the career determination process that starts with the choice of medical school. These factors include personal interest/ability, sustainable lifestyles or flexible work schedules, income, family, role models, duration of education,^8^ gender,^1^,^9^ working conditions, academic career opportunities, professional satisfaction, and malpractice.^10^ No studies specifically investigating the effect of PC on the career plans of medical students could be found in the existing literature. This study aimed to evaluate the impact of PC on the career choices of intern doctors and to provide important information for policy makers and medical educators in health workforce planning.

## METHODS

This cross-sectional study was conducted in Erzincan Province in eastern Turkiye. There is one medical faculty in Erzincan Province. The population of the study consisted of Erzincan Binali Yıldırım University Faculty of Medicine senior year students (interns). No sample was selected, and it was planned to reach the entire population (138 students). A total of 129 (93.5%) interns voluntarily participated in the study. The intern doctors were reached in the clinics where they were doing their internship. Following the information provided by the researchers, the questionnaire forms were filled out by the students. During the completion of the questionnaires under observation, the questions of the students were answered by the researchers. The questionnaire forms application was completed between April 15^th^ and November 15^th^ 2023.

### Ethical approval:

This research was approved by the Erzincan Binali Yıldırım University Health and Sports Ethics Committee (Date: March 31, 2023 Number: 03/13). The participating students were briefed on the study’s objectives, and their consent was obtained prior to their involvement.

In this study, a questionnaire form based on the literature and prepared by the researchers and the Commitment to Profession of Medicine Scale (CPMS) were used as the data collection tools. The questionnaire consisted of 16 closed-ended questions. The questionnaire consists of two parts. In the first part, there are 11 questions evaluating the descriptive characteristics of the students such as age, gender, parental education level, family income, where they have lived for the longest time, foreign language proficiency, whether they have repeated the semester/internship, whether there are doctors/specialists in their families and whether they have chosen the medical faculty willingly. The second part consists of five questions assessing career preferences such as the desire to become a specialist, the status of deciding on the speciality department, the speciality preference, the preferred place to work after compulsory service, and whether their speciality preferences changed according to the first years of their education. The risk assessment of specialties was conducted based on the risk categories outlined in the Communiqué on Procedures and Principles Regarding Institutional Contribution in Compulsory Liability Insurance for Medical Malpractice^11^ which is currently in effect in Turkiye. According to this communiqué, specialties are categorized into four groups, with the first group representing the lowest risk and the fourth group representing the highest. The CPMS was developed by Aytuğ Koşan and Toraman in 2020.^12^

The validity and reliability study was conducted with Turkish medical students and the scale consists of 9 items with a 5-point Likert scale (Strongly Disagree=1, Disagree=2, Partially Agree=3, Agree=4, and Strongly Agree=5). The scale is unidimensional and a total score can be obtained from nine items in the scale. The maximum score that can be obtained from the scale is 45 and the minimum score is nine. A high score on the scale indicates a strong commitment to the medical profession. During the scale’s development, its construct validity was established, and the reliability coefficient was calculated as α= 0.876. Permission to use the scale was obtained from the scale’s developers via email.

Data were analyzed using IBM SPSS Statistics for Windows 25.0 (IBM Corp., Armonk, NY, USA). The normality assumption of the data was evaluated using the Shapiro–Wilks or Kolmogorov–Smirnov test and homogeneity of the variances was evaluated using the Levene’s test. Descriptive statistics were presented as the mean ± standard deviation (SD) for variables with normal distribution, median (min-max) for variables with non-normal distribution, and frequency and percent (%) for the categorical variables. Considering the assumption of normality, comparisons between two groups were evaluated by Student T test comparing the mean values between groups and Mann Whitney U test comparing the median values. More than two groups were analysed with Kruskal Wallis test in terms of median values. Dunn Bonferonni test was used as a multiple comparison test for Kruskal Wallis test. Results were considered statistically significant at p<0.05.

## RESULTS

### Descriptive findings and pc levels according to descriptive findings:

A total of 129 students participated in the study, with a mean age of 24.3±1.2 years, of whom 50.4% were female. 34.9% of the student`s mothers were primary school graduates, 48.8% of the students’ fathers were university graduates and 47.3% of the students defined their family income as equal to their expenses. The proportion of students with a doctor in their families was 51.9% and the proportion of students with a specialist doctor was 43.4%. Foreign language level was defined as intermediate by 52.7% of the students. 24.8% of the students had a semester/internship loss. 87.6% of the students stated that they lived in the urban area for a long time. Additionally, 88.4% of the students voluntarily chose medical school. The descriptive characteristics of the students are presented in Table-I. The mean CPMS score of the participants was 31.3±6.5. The CPMS score was 31.4±6.5 for the females and 31.2±6.5 for the males, and there was no statistical difference between them (p>0.05). The CPMS scores of the students who chose medical school voluntarily (median= 31, min-max=19-45) were higher than those of the students who did not (median=26, min-max=17–32) and the difference between them was statistically significant (U=403.0, p=0.001). There was no statistical difference between the CPMS scores with respect to the other descriptive characteristics (p>0.05).

**Table-I:** Descriptive characteristics of the students (n=129).

Variables	Number (n)	Percentage (%)
***Age (Mean±SD)***		
Female 24.2±1.2		
Male 24.4±1.3		
***Sex***		
Female	65	50.4
Male	64	49.6
***Mother’s education status***		
Illiterate	6	4.7
Literate	6	4.7
Primary education	45	34.9
High school	29	22.4
University	43	33.3
***Father’s education status***		
Illiterate	4	3.1
Literate	2	1.6
Primary education	21	16.3
High school	39	30.2
University	63	48.8
***Family income***		
Income less than expenditure	8	6.2
Income equal to expenditure	61	47.3
Income more than expenditure	60	46.5
***Place lived in the longest***		
Rural area	16	12.4
Urban area	113	87.6
***Foreign language***		
Bad	31	24.0
Medium	68	52.7
Good	21	16.3
Very good	9	7.0
***Term/Internship repeat status***		
Yes	32	24.8
No	97	75.2
***Have a doctor in the family***		
Yes	67	51.9
No	62	48.1
***Have a specialist doctor in the family***		
Yes	56	43.4
No	73	56.6
***Voluntarily chose medical school***		
No	15	11.6
Yes	114	88.4

### Career plans and pc levels according to career plans:

Of the students, 93% expressed a desire to specialize, with 37% opting for internal medical sciences and another 37% selecting specialties from the fourth risk group. The specialization areas preference of 62.5% of the students changed according to the first years of medical school. About 82.9% of the students were planning to work in Turkey after compulsory service. The PC scores of the students who wanted to become specialists were found to be statistically significantly higher than the PC scores of the students who did not want to become specialists (p= 0.040). PC scores showed a statistically significant difference between risk groups according to specialty risk assessment (p=0.021). This difference was due to the difference between the fourth risk group with the highest PC score and the second risk group with the lowest PC score. The PC scores of the students who wanted to work in Turkey after compulsory service were higher than the PC scores of the students who wanted to work abroad and the difference was statistically significant (p= 0.047). Table-II displays the CPMS scores based on the students’ career preferences. According to specialties, neurosurgery was preferred by students with the highest CPMS score and family medicine was preferred by students with the lowest CPMS score. Table-III displays the CPMS scores categorized by specialty areas.

**Table-II:** Veocational commitment scores of the students according to their career preferences.

Variables	Total	CPMS (Mean±SD)	CPMS Median (min–max)	p-value
***Want to be a specialist (n=129)***				
No	9 (7.0)	26.8±5.5	27 (19–36)	0.040^Φ^
Yes	120 (93.0)	31.6±6.5	31 (17–45)	
***Decided on the area of specialization(n=120)***				
Haven’t made a decision yet	39 (32.5)	30.3±5.8	30 (19–44)	0.110^ψ^
Made a decision	81 (67.5)	32.3±6.7	32 (17–45)	
***Area of specialization (n=81)***				
Internal Medical Sciences	30 (37.0)	30.8±6.6	31 (17–43)	0.115^ψ^
Surgical Sciences	51 (63.0)	33.2±6.6	32 (21–45)	
***Specialization risk groups (n=81)***				
Group 1	–	–	–	
Group 2	14 (17.3)	28.6±7.6	28 (17–43)^a^	0.021^ω^
Group 3	37 (45.7)	31.7±4.9	32 (20–41)^ab^	
Group 4	30 (37.0)	34.8±7.3	33.5 (21–45)^b^	
***Work location preference after obligatory service (n=129)***				
Turkiye	107 (82.9)	31.8±6.7	31 (17–45)	0.047^Φ^
Abroad	22 (17.1)	28.7±4.9	29 (21–38)	
***Change in specialization area preference compared to the first year of medical school (n=120)***				
No	45 (37.5)	33.0±6.6	32 (17–45)	0.085^ψ^
Yes	75 (62.5)	30.9±6.3	30 (19–45)	

Φ Mann–Whitney U test,

ψ T test,

ω Kruskal–Wallis test, Statistical significance was p< 0.05. ^a,b^Groups indicated with the same letter were statistically similar, while there was a statistically significant difference between groups labeled with different letters at the 0.05 significance level. CPMS: Commitment to Profession of Medicine Scale.

**Table-III:** Students’ PC scores according to their specialization area preferences (n=81).

Areas of specialization	n (%)	CPMS (Mean±SD)
***Specialization risk group 2***		
Family medicine	4 (4.9)	26.3±9.3
Dermatology	4 (4.9)	26.5±2.5
Sports medicine	4 (4.9)	28.8±8.4
Physical therapy and rehabilitation	2 (2.5)	38.0±7.1
***Specialization risk group 3***		
Radiology	5 (6.2)	29.6±6.8
Ophthalmology	11 (13.6)	29.7±3.6
Otolaryngology	7 (8.6)	31.1±5.1
Mental health and diseases	1 (1.2)	32.0
Pediatrics	4 (4.9)	32.5±5.2
Cardiology	4 (4.9)	35.0±3.5
Urology	3 (3.7)	35.0±7.2
Infectious diseases	2 (2.5)	36.0±1.4
***Specialization risk group 4***		
Obstetrics and gynecology	6 (7.5)	31.5±7.2
Orthopedics and traumatology	10 (12.4)	32.1±8.0
Plastic surgery	2 (2.5)	33.0±2.8
Anesthesiology	2 (2.5)	33.5±3.5
General surgery	3 (3.7)	39.0±9.5
Emergency	4 (4.9)	39.5±4.5
Cardiovascular surgery	2 (2.5)	40.5±3.5
Neurosurgery	1 (1.2)	44.0

CPMS: Commitment to Profession of Medicine Scale.

## DISCUSSION

The findings herein revealed that students who chose medical school voluntarily exhibited higher levels of PC. Students’ autonomous decision-making ability positively affects professional commitment. This outcome aligns with previous studies conducted among Turkish medical students.^12^,^13^ No significant difference was observed in the CPMS scores based on the sex of the students in this study. Previous studies on medical students in Turkiye have shown higher levels of PC among females^5^,^13^, while a study on nurses in Iran found no sex-based difference.^14^ An important finding of the current study was that students aspiring to become specialists had higher levels of PC. However, the fact that very few students in the study chose to become general practitioners and that these students had lower CPMS scores pointed to another dimension of the problem.

This outcome may negatively impact primary and preventive health services in Turkiye. Previous studies have also shown that only a small proportion of Turkish medical students want to work as general practitioners, while the majority prefer to become specialists.^10^,^15^ Similar to Turkiye, the recruitment of primary care doctors is a challenge faced by many countries worldwide.^16^ Health policies should be designed to positively influence doctor prestige and PC, particularly in primary care. The present study indicated that students who favored specialties in the highest occupational risk group exhibited higher CPMS scores. In recent years in Turkey, medical students do not prefer higher risk surgical specialities, possibly due to factors such as increased malpractice lawsuits, long working hours, difficulties in work-life balance and workplace violence.^15^,^17^ However, the present findings suggest that the difficulty and risks associated with a specialty do not significantly alter the career plans of students with high levels of PC. This result emphasizes the importance of training physicians with a high level of professional commitment for the future of these important specialities, which are increasingly less preferred in Turkey.

When examining the specialty preferences of the students, it is notable that those who favored family medicine had lower levels of PC, and none of the students chose public health as a specialty. This problem is particularly significant as it may adversely impact primary health care services. The lack of practical exposure to public health and family medicine until the final year of medical education in Turkiye may have adversely impacted students’ preferences and levels of PC. Studies on medical students in Saudi Arabia^18^ found a similar lack of interest in public health and basic sciences, while in Pakistan^19^ there was less enthusiasm for family medicine and public health. Integrating primary care and field training into the early years of medical education, along with providing students with exemplary role models in primary care, can enhance their motivation and levels of PC. The limited interest in fundamental sciences and specific clinical disciplines as career options highlights the necessity for academic acknowledgment and support for these areas via medical education and health policies.

Students who expressed a desire to work in Turkiye after their obligatory service showed higher levels of PC. Similar findings were reported in a study by Erbir^13^ on medical students in Turkiye. Doctor migration, a challenge also observed in other countries^20^,^21^, has become a significant health workforce issue in Turkiye in recent years. Studies conducted in Turkiye have identified several key factors contributing to doctor migration, including diminishing doctor prestige, instances of violence against doctors, unfavorable working conditions^22^ and inadequate wages.^22^,^23^ Despite these problems perceived by doctors in Turkiye, students with high levels of PC do not consider migrating to another country. Policy makers should take action to improve doctors’ working conditions and raise PC if they want to reduce doctor migration.

### Limitations:

Since the research was conducted in a single medical faculty, the results do not represent all medical students in the country. It is recommended that the study be conducted in different medical faculties with larger sample sizes. Another limitation of this study was that it was conducted only with students in their final year of medical school. Implementing the study across all medical school classrooms could yield more insights on the evolution of PC during academic training.

## CONCLUSIONS

This study showed that the degree of professional commitment can affect medical students’ career goals. The desire to become a specialist physician, preference for riskier specialities and the desire to work in Turkey after graduation were found to be associated with high levels of PC. According to the results of the study, policy makers should consider the impact of MA on physicians’ career plans in order to create a multifaceted strategy for the health workforce. Furthermore, medical educators should develop educational programme aiming to improve their students’ PC while planning their curricula. Future studies should investigate the impact of PC on long-term career intentions across a wider range of fields and different student demographics.

### Authors’ Contribution:

**ST and SH:** Designed the study, data collection, statistical analysis, manuscript writing, review and approval of manuscript.

All authors are accountable for the integrity of work.
